# Detection of Myopia from Colour Fundus Photographs Using YOLO-Based Computer-Vision Models: A Comparison with Expert Ophthalmologists

**DOI:** 10.3390/jcm15166209

**Published:** 2026-08-11

**Authors:** Nicola Rizzieri, Luca Dall’Asta, Maris Ozolinš

**Affiliations:** 1 Department of Optometry and Vision Science, The Faculty of Science and Technology, University of Latvia, Jelgavas Street 1, LV-1004 Riga, Latvia; maris.ozolins@lu.lv; 2 LIFE Srl, Research and Development, IT-70100 Bari, Italy; dallas.luca@gmail.com; 3 Institute of Solid State Physics, University of Latvia, Kengaraga Street 8, LV-1063 Riga, Latvia

**Keywords:** myopia, fundus photography, YOLO, computer vision, artificial intelligence, screening tools

## Abstract

**Objectives:** The purpose of this study was to evaluate the ability of computer vision models to detect myopia from standard colour fundus photographs and to compare their diagnostic performance with that of experienced ophthalmologists. **Methods:** A previously published dataset of 324 retinal fundus images labelled as myopic or non-myopic based on cycloplegic refraction as used for model training and internal validation. Images were acquired using a non-mydriatic 45° fundus camera. Final model evaluation was performed on an independent test set of 50 images from different patients who were not included in the original dataset. YOLOv8 and YOLOv11 variants were trained for binary classification. Internal validation used patient-level cluster bootstrap confidence intervals, whereas image-level bootstrap confidence intervals were estimated for the independent test set. Pairwise model comparisons were adjusted using the Holm–Bonferroni correction. Five experienced ophthalmologists independently classified the test set, and their consensus was compared with the selected YOLO models using DeLong’s and exact McNemar tests. **Results:** Internal validation identified YOLOv8-m and YOLOv11-n as the best-performing models according to a predefined composite score used exclusively for model selection. On the independent test set, YOLOv11-n achieved the highest area under the curve (AUC = 0.889), followed by YOLOv8-m (0.806), although the difference was not statistically significant (DeLong test, *p* > 0.05). The clinical consensus achieved an AUC of 0.832, with no significant difference compared with either model. Exact McNemar testing likewise revealed no statistically significant differences in paired classification outcomes between either AI model and the clinical consensus. Limitations include the small, single-centre, class- and age-imbalanced dataset and the limited number of expert observers. **Conclusions:** Although neither YOLOv8-m nor YOLOv11-n showed statistically significant differences from the clinical consensus on this independent test set, these findings should be interpreted cautiously given the relatively small, single-centre study population. Larger multicentre studies with independent external validation are warranted to confirm the generalisability, robustness, and potential role of clinician-driven computer vision models as decision support tools for myopia screening.

## 1. Introduction

Myopia is one of the most prevalent refractive errors worldwide and represents a major public health concern. While affecting approximately 20–30% of Western youth, the condition reaches epidemic proportions in East Asia, where figures exceed 70% among the school-aged population [1,2,3]. A landmark systematic review and meta-analysis estimated that by 2050, nearly half the global population—approximately 4.8 billion people—is expected to be myopic, with one billion affected by high myopia [4]. Beyond simple refractive error, these high-grade forms predispose patients to vision-threatening pathologies, including retinal detachment, myopic maculopathy, glaucoma, and cataracts, ultimately imposing a profound socioeconomic burden and diminishing quality of life [4,5,6,7,8,9].

Standard clinical diagnosis relies on distance visual acuity and refractive measurement, with cycloplegic refraction remaining the gold standard to eliminate accommodative bias, especially in younger cohorts [10,11]. While essential for precise optical quantification, these methods are distinct from retinal imaging. Conventionally, colour fundus photography is reserved for monitoring structural complications, such as chorioretinal atrophy or lacquer cracks, rather than for primary diagnosis [12]. Identifying non-pathological (low-to-moderate) myopia from such images remains a formidable task for human observers, as these eyes often lack overt funduscopic abnormalities. However, subtle textural, vascular, or spatial patterns associated with myopia, though imperceptible to the human eye, may still be encoded within the retinal architecture [13].

Recent advances in artificial intelligence (AI), particularly in deep learning (DL) and computer vision, have revolutionised medical imaging, enabling the automated detection of conditions such as diabetic retinopathy, glaucoma and age-related macular degeneration with expert-level accuracy [14,15,16]. While previous DL research has focused on identifying pathological myopia through lesion recognition [17,18], there is growing evidence that latent features associated with refractive status can be extracted directly from pixel data [19].

This study explores the frontier of this technology by investigating whether state-of-the-art object detection and classification frameworks can discern myopia—specifically non-pathological forms (i.e., eyes with myopic refractive error without clinically evident ocular comorbidities)—from standard 45° fundus images. We focus on the “You Only Look Once” (YOLO) family of architectures, which offers a robust balance of efficient feature extraction and multi-scale representation. A distinctive aspect of our approach is the utilisation of the Roboflow application programming interface (API) [20], a platform that democratises AI implementation by facilitating model training and deployment for clinical researchers without extensive programming expertise.

Building upon prior benchmarks established with YOLOv8 [21], the present research further extends its investigation by evaluating the performance of the latest YOLOv11 iteration [22]. By benchmarking the best models against a panel of experienced ophthalmologists, we aim to determine whether computer vision models could achieve or outperform human diagnostic performance in the binary classification of myopia versus non-myopia from standard colour fundus pictures. This comparison was intended to provide clinical context for the potential role of AI as a complementary decision support tool rather than as a replacement for comprehensive ophthalmic assessment.

## 2. Materials and Methods

### 2.1. Study Population and Data Acquisition

For this retrospective and observational study, aimed at developing and validating a supervised machine learning algorithm for binary classification, we used the retinal fundus image dataset previously collected and described in our earlier publication [21] exclusively for model training and internal validation. To ensure an independent assessment of diagnostic performance, a separate test dataset, consisting of retinal fundus images from different patients not included in the original dataset, was subsequently collected and used exclusively for the final evaluation and comparison with expert ophthalmologists.

A total of 338 retinal fundus images were collected from 169 Caucasian patients, aged 7–84 years, who attended an optometry and contact lens practice in Northern Italy for routine eye examinations (Supplementary Table S1) from March to August 2023. All participants were evaluated by a licensed optometrist during standard clinical visits. The examination protocol included assessment of medical history and visual complaints, measurement of distance visual acuity (unaided and best corrected), static retinoscopy, subjective refraction, slit-lamp biomicroscopy, and colour fundus photography. Objective refractive error was measured under cycloplegia using an Oculus Myopia Master (OCULUS Optikgeräte GmbH, Wetzlar, Germany). Cycloplegia was induced with 1% tropicamide administered as three drops at 15 min intervals under the supervision of an ophthalmologist. Objective refraction was performed approximately 60 min after the first instillation. Adequate cycloplegia was confirmed by the absence (or marked reduction) of the pupillary light reflex together with adequate pupillary dilation before refraction.

Only eyes without concomitant ocular pathology were included. Patients with retinal disease, optic nerve disorders, media opacities, previous ocular surgery, or any other ocular condition potentially affecting fundus appearance were excluded, ensuring that myopia was the only ocular condition present in the study population. The full study population description is available in Table S1 of the Supplementary Materials.

All retinal images were acquired by the same experienced optometrist using a non-mydriatic Optopol REVO FC 80^®^ fundus camera (Optopol Technology, Zawiercie, Poland; software version 11.5.0). The device is equipped with a 12.3-megapixel sensor and provides a field of view of 45 ± 5°. Image acquisition was performed in a darkened room using the central fundus photography mode, with automatic flash intensity and gain level adjustment. Fundus photographs were captured in PNG format with RGB colour encoding and subsequently exported from the device software for further analysis.

Only images of sufficient quality were included in the study and retrospectively analysed. Inclusion criteria required images to be well-centred, sharp, and free from motion blur, illumination artefacts, or obscuring media opacities. Each image was reviewed by the optometrist to ensure that key anatomical structures, including the macula, optic disc, and retinal vasculature, were clearly visible and in focus. Images that were blurred, poorly illuminated, off-centre, or affected by artefacts were excluded from the dataset.

### 2.2. Data Preparation

All selected fundus images were anonymised prior to analysis. Images were then divided into two datasets according to the objective spherical equivalent refraction (SER) of each eye. SER was calculated using the following standard formula:

$$SER=SF+\;\frac{CYL}{2}$$

where *SF* represents the spherical power and *CYL* represents the cylindrical power of the corrective lens.

In accordance with the International Myopia Institute (IMI) definition, myopia was defined as a refractive condition in which SER is ≤−0.50 dioptres (D) [11]. Eyes with SER ≤ −0.50 D were classified as myopic, whereas eyes with SER > −0.50 D were classified as non-myopic.

Based on these criteria, 124 patients were diagnosed as myopic (74 females; mean age ± standard deviation: 26.74 ± 13.78 years), while 45 patients were classified as non-myopic (27 females; mean age ± standard deviation: 40.33 ± 17.74 years). Recruitment of non-myopic subjects proved particularly challenging, since most patients attending the clinic were myopic. This also reflects the fact that the clinic is highly specialised in myopia and myopia management, which partly explains why the mean age of the myopic group was lower than that of the non-myopic group.

Following image quality assessment, 14 out-of-focus fundus photographs corresponding to 7 participants were excluded. The final development dataset therefore comprised 324 retinal fundus photographs from 162 participants (see Supplementary Figure S1), comprising 238 images from the myopic group and 86 images from the non-myopic group. Both right and left eyes were included. The mean SER of the myopic eyes was −3.19 ± 2.84 D, whereas the mean SER of the non-myopic eyes was +0.92 ± 1.32 D (Supplementary Table S1). The development dataset was partitioned at the patient level into a training set (122 participants; 244 images) and an internal validation set (40 participants; 80 images). Final model evaluation was performed on a separate independent cohort comprising 50 retinal fundus photographs from 50 different patients who were not included in the development dataset.

Written informed consent was obtained from all participants or their legal guardians prior to participation. The study was conducted in accordance with the principles of the Declaration of Helsinki.

### 2.3. Dataset Splitting and Augmentation

Both YOLOv8 and YOLOv11 require class-labelled images to perform image-level classification tasks. Therefore, each fundus image was manually class-labelled as either myopic or non-myopic according to the SER-based classification. Annotation was performed using freely available online annotation software [20], and the labelled datasets were subsequently downloaded for model development.

To prevent data leakage and ensure an unbiased evaluation, dataset partitioning was performed at the patient level, ensuring that both right and left eyes from the same individual were assigned to the same subset. The resulting dataset was then divided into training and internal validation sets before applying any data augmentation procedures. Data augmentation was performed exclusively on the training set to mitigate overfitting and improve model generalisation, whereas the internal validation set consisted solely of previously unseen, non-augmented images. For the final evaluation, an independent test set was assembled using retinal fundus images from different patients who were not included in the original development dataset but were recruited from the same clinical centre. The independent test set contained a single fundus image from one eye of each patient. Therefore, no patient contributed more than one image, and the individual image represented the statistical unit for all analyses performed on the independent test set.

Following data augmentation applied exclusively to the training subset, model development was performed using 569 augmented training images and 80 original, non-augmented validation images. In our previous publication [21], the remaining 50 images from the same dataset were used as an internal test set. In the present study, this internal test set was replaced by a newly collected independent test set consisting of 50 retinal fundus photographs obtained from different patients who were not included in the original dataset. Consequently, the independent test set used in the present study contains no images or participants from the previous publication and was used exclusively for the final performance evaluation.

Early stopping was implemented such that training was terminated if no improvement in validation performance was observed for ten consecutive epochs. All experiments were conducted by an optometrist with basic knowledge in computer programming, on a workstation equipped with an Intel^®^ Core™ i7 processor, 64 GB RAM, and an NVIDIA^®^ GeForce RTX™ 3070 Ti GPU with 8 GB of dedicated memory. All the steps were supervised by an experienced computer scientist to ensure correctness and methodology.

### 2.4. YOLOv8 and YOLOv11 Architectures and Training Details

In this study, two models from the YOLO family were employed to perform binary image-level classification of myopia from retinal fundus photographs. Although originally designed for object detection, modern YOLO implementations support classification tasks by leveraging powerful backbone networks capable of extracting hierarchical and multi-scale visual features [23].

YOLOv8 models were implemented using the Ultralytics framework [24]. Five classification variants differing in model complexity and number of parameters were evaluated, allowing analysis of the trade-off between computational efficiency and classification performance. YOLOv8 adopts a convolutional backbone with Cross Stage Partial (CSP)-inspired modules combined with a decoupled head design, which improves feature representation and training stability. This architecture enables efficient learning from relatively small datasets while maintaining strong generalisation capabilities [25].

To extend previous findings, the present work additionally evaluated the recently introduced YOLOv11 architecture [26,27]. YOLOv11 builds upon earlier YOLO versions while incorporating architectural refinements aimed at improving feature extraction, optimisation efficiency, and inference accuracy. These include improved backbone feature fusion and more effective gradient propagation, making YOLOv11 particularly suitable for fine-grained visual classification tasks. To the best of our knowledge, this is one of the first studies to apply YOLOv11 for the binary classification of myopia from fundus images.

### 2.5. Training Procedures

All image classification models (YOLOv8 and YOLOv11) were developed using the no-code training environment provided by [20], which implements the Ultralytics image classification framework (version 8.3.70) [27]. To ensure a fair comparison between the two model families, identical pre-processing procedures, data augmentation strategies, optimisation settings, and training protocols were applied across all experiments, with the network architecture representing the only experimental variable.

Transfer learning was employed by initialising each model with ImageNet-pretrained weights prior to fine-tuning on the study dataset. During training, all fundus photographs were automatically resized to 640 × 640 pixels. Image augmentation was performed using the default Roboflow/Ultralytics classification pipeline, including RandAugment, random horizontal flipping (probability = 0.5), random scaling (±50%), random translation (10%), HSV colour augmentation, and random erasing (probability = 0.4). Automatic Mixed Precision (AMP) was enabled to improve computational efficiency during training.

All models were trained for a maximum of 100 epochs using a batch size of 4. Optimisation was performed using the default automatic optimiser selection implemented by the Ultralytics framework, with an initial learning rate of 0.01, momentum of 0.937, weight decay of 5 × 10^−4^, and a warm-up period of 3 epochs. To improve reproducibility, all experiments were conducted using a fixed random seed (seed = 0), with deterministic training enabled.

No explicit class imbalance mitigation strategies, such as class weighting, oversampling, undersampling, or focal loss, were applied during model training. Instead, the original class distribution of the dataset was preserved throughout the training process using the standard Roboflow no-code workflow. This approach was intentionally adopted to evaluate the performance of a clinically applicable no-code artificial intelligence workflow under conditions representative of routine clinical practice, rather than to maximise classification performance through additional algorithmic interventions.

Model predictions were generated according to the standard implementation of the Ultralytics classification framework, whereby the predicted class corresponded to the category with the highest posterior probability produced by the softmax output layer. In the binary classification setting adopted in this study, this decision rule is mathematically equivalent to applying a probability threshold of 0.50; therefore, no additional confidence threshold was specified or optimized.

To facilitate reproducibility, all training hyperparameters, augmentation settings, and training logs were automatically recorded by the Roboflow/Ultralytics framework and archived together with the trained model checkpoints. The trained model weights, training configuration files, and anonymised datasets supporting the findings of this study are available from the corresponding author upon reasonable request, subject to institutional regulations and ethical approval.

Model performance was monitored using the internal validation set, and final evaluation was conducted exclusively on the independent test set (50 completely new images).

### 2.6. Model Explainability Analysis

To explore the image regions contributing to model predictions, Gradient-weighted Class Activation Mapping (Grad-CAM) [28] was applied to selected correctly and incorrectly classified fundus images for both YOLOv8 and YOLOv11 models on the independent test set only. Grad-CAM heat maps were generated from the final convolutional layers of the classification backbone to visualise spatial activation patterns associated with myopia predictions. The heat maps were qualitatively inspected to assess whether model attention converged on specific retinal anatomical structures or regions.

### 2.7. Clinical Expert Protocol

To benchmark the performance of the deep learning models against human expertise, a panel of five experienced ophthalmologists was recruited to independently classify the retinal fundus images included in the independent test set. All participating clinicians had at least ten years of clinical experience and were primarily involved in the management of myopic patients and in medical retina practice. The same subset of colour fundus photographs used to evaluate the YOLOv8 and YOLOv11 models (i.e., the independent test set) was also used for the human expert assessment, ensuring methodological consistency and enabling a direct comparison between artificial intelligence models and human observers. Each ophthalmologist was asked to classify each image as either myopic or non-myopic based solely on visual inspection of the retinal photograph. To standardise the evaluation procedure, the images were uploaded to a Google Forms questionnaire and presented in randomised order. Each clinician accessed the questionnaire through a dedicated web link and completed the evaluation remotely using their own computer. No additional clinical information, such as refractive measurements, patient age, visual acuity, or medical history, was provided in order to replicate the information available to the deep learning models and to avoid potential sources of bias.

All ophthalmologists were blinded to the ground-truth refractive status of the eyes and to the predictions generated by the deep learning models. No time constraints were imposed, and participants were instructed to rely exclusively on their professional judgment of retinal features potentially associated with myopia. Individual classifications were recorded automatically through the questionnaire platform and subsequently compared with the reference labels derived from cycloplegic refraction, which served as the sole criterion to define the binary ground truth (myopic vs. non-myopic). Importantly, ophthalmologists were not asked to estimate or infer the spherical equivalent refraction value but only to perform a categorical classification of refractive status. Accordingly, the performance evaluation of both the human experts and the YOLO-based models was conducted exclusively at the level of binary discrimination between myopia and non-myopia.

Individual diagnostic labels were aggregated into a consensus prediction to provide an overall estimate of human diagnostic performance; a sample was classified as myopic if a majority (≥3 out of 5 experts) identified the condition. This approach reduces inter-observer variability and allows robust comparison between AI models and human observers [29].

### 2.8. Performance Metrics

The performance of the deep learning models and human experts was evaluated using a comprehensive set of standard metrics for binary classification. For each model and each ophthalmologist, the following measures were computed based on the confusion matrix [30]:

- Accuracy (ACC) is defined as the ratio of correct predictions to the total predictions made, as illustrated in Equation (1).
- Precision (P) assesses the ratio of true positives in all positive predictions, computed using Equation (2).
- Sensitivity (S) determines the ratio of true positives in all actual positives. S measures the model’s effectiveness in identifying all instances of a class, as illustrated in Equation (3).
- Specificity (Sp) calculates the ratio of true negatives in all negative predictions, as shown in Equation (4).
- The F1 score represents the harmonic mean of P and S, providing a balanced evaluation of the model’s accuracy by taking into account false positives and negatives, as demonstrated in Equation (5).
- The receiver operating characteristic (ROC) curve is a graphical representation of a classification model’s performance across all classification thresholds. It shows the trade-off between the true positive rate (TPR) and the false positive rate (FPR). TPR is also known as recall or sensitivity (Equation (3)). The FPR is the ratio of incorrectly identified negative instances to the total actual negative instances, illustrated in Equation (6). As the classification changes, both the TPR and FPR change, and plotting them forms the ROC curve and the corresponding area under the curve (AUC), computed from the continuous probability outputs of the deep learning models.

(1) $$Accuracy=\frac{TP+TN}{TP+TN+FP+FN}$$

(2) $$Precision=\frac{TP}{TP+FP}$$

(3) $$Recall\;or\;Sensitivity\;or\;TPR=\frac{TP}{TP+FN}$$

(4) $$Specificity=\frac{TN}{TN+FP}$$

(5) $$F1\;score=\frac{TP}{TP+\frac{1}{2}(FP+FN)}$$

(6) $$FPR=\frac{FP}{FP+TN}$$

where TP is true positive, TN is true negative, FP is false positive, and FN is false negative.

Particular emphasis was placed on sensitivity and Matthews correlation coefficient (MCC), which provides a balanced measure of classification quality even in the presence of class imbalance and ranges from −1 (total disagreement) to +1 (perfect agreement) [31]. Sensitivity was prioritised because missing myopic cases (false negatives) may delay diagnosis and management, potentially increasing the risk of long-term ocular complications. MCC was included to provide a robust global assessment of classification performance that accounts for all four components of the confusion matrix and mitigates the influence of class imbalance. The formula for the MCC is shown in Equation (7).

(7) $$MCC=\frac{\left(TP\times TN\right)-(FP\times FN)}{\sqrt{\left(TP+FP)(TP+FN)(TN+FP)(TN+FN\right)}}$$

The negative predictive value (NPV) is defined as the proportion of true non-myopic cases among all images classified as non-myopic [32]. The higher the NPV, the more reliable the test is at ruling out the disease. NPV is described by Equation (8):

(8) $$NPV=\frac{TN}{TN+FN}$$

For the clinical evaluation, performance metrics were computed individually for each ophthalmologist and for the clinical consensus, defined using majority voting (at least three concordant classifications out of five). For ROC analysis of the consensus, a continuous score was defined as the proportion of clinicians classifying each image as myopic.

### 2.9. Statistical Analysis

Statistical analyses were performed using Python (version 3.12.7) within the JupyterLab environment (version 4.2.5), employing standard scientific libraries including NumPy, pandas, SciPy, scikit-learn, and statsmodels.

For the internal validation dataset (60 myopic and 20 non-myopic eyes), to quantify the uncertainty of performance estimates, 95% confidence intervals were computed using a paired cluster bootstrap procedure (2000 iterations), with resampling performed at the patient level to preserve the correlation between fellow eyes. Pairwise comparisons between corresponding YOLOv8 and YOLOv11 model variants (nano, small, medium, large, and extra large) were conducted using the same paired cluster bootstrap framework. For each comparison, the observed difference in each performance metric was calculated from the original validation dataset. Bootstrap resampling was then used to estimate the sampling distribution of the difference, from which percentile-based 95% confidence intervals were derived. Empirical two-sided *p*-values were obtained by centring the bootstrap distribution on the observed difference and calculating the proportion of bootstrap replicates with an absolute deviation equal to or greater than the observed effect under the null hypothesis. To control for multiple comparisons, *p*-values were adjusted using the Holm–Bonferroni correction, and adjusted *p*-values < 0.05 were considered statistically significant.

Model selection was performed exclusively on the internal validation dataset to preserve the independence of the held-out test set for final performance evaluation. To identify the most clinically suitable model within each YOLO architecture family, a predefined weighted composite performance score was calculated by combining sensitivity (45%), Matthews correlation coefficient (30%), and F1 score (25%). These weights were selected a priori to reflect the clinical priorities of a screening-oriented application, where maximising sensitivity is essential while maintaining balanced overall classification performance. Prior to weighting, each performance metric was independently min–max normalised across the evaluated model variants to a common scale ranging from 0 to 1, thereby ensuring comparability between metrics. The composite score was then calculated as the weighted sum of the normalised metrics, and the highest-scoring YOLOv8 and YOLOv11 variants were selected for subsequent evaluation on the independent held-out test set and comparison with the ophthalmologist consensus.

For the independent test set (35 myopic and 15 non-myopic eyes), uncertainty in performance estimates was quantified by calculating 95% confidence intervals using a paired non-parametric bootstrap procedure with 2000 resamples. Because the independent test dataset contained a single fundus image from one eye of each patient, bootstrap resampling was performed at the patient level, with one independent fundus image available for each patient. ROC curves and AUC values were computed for the selected YOLOv8 and YOLOv11 models and for the clinical consensus. Differences in AUC were assessed using DeLong’s test for correlated ROC curves, accounting for the paired nature of predictions generated on the same images. To evaluate differences at the decision level, the exact McNemar test was applied to paired binary predictions between YOLOv8 and YOLOv11; YOLOv8 and the clinical consensus; and YOLOv11 and the clinical consensus. Because the exact binomial version of the test was used, only the discordant counts (b and c) and the corresponding exact two-sided *p*-values are reported. Inter-observer agreement among the five ophthalmologists was quantified using Fleiss’ kappa (κ), and pairwise agreement between observers was evaluated using Cohen’s kappa. All statistical tests were two-sided, and statistical significance was defined as *p* < 0.05 unless otherwise specified.

## 3. Results

### 3.1. Internal Validation

Overall, models demonstrated high sensitivity for the detection of myopia, indicating a strong ability to correctly identify myopic eyes. However, relevant differences were observed across architectures and model scales in terms of overall classification balance, particularly when considering sensitivity, F1 score, and MCC. Confusion matrices for all YOLO model variants, including TP, TN, FP, and FN values, are shown in the Supplementary Materials (Figures S2 and S3).

Table 1 summarises the performance metrics of all YOLOv8 and YOLOv11 variants on the internal validation set, reported as mean values (central estimate from the original dataset) with corresponding 95% bootstrap CIs (calculated using bootstrap resampling with 2000 iterations).

Overall, the YOLOv8 models demonstrated heterogeneous performance on the validation dataset. According to the predefined composite score, YOLOv8-m achieved the highest overall ranking, combining excellent sensitivity (0.967, 95% CI: 0.917–1.000), the highest F1 score (0.885, 95% CI: 0.818–0.939), and a high MCC (0.434, 95% CI: 0.147–0.661). Although YOLOv8-n achieved the highest specificity (0.550, 95% CI: 0.308–0.773) and the highest MCC (0.449, 95% CI: 0.186–0.668), its slightly lower F1 score resulted in a lower composite score. In contrast, YOLOv8-l showed very high specificity (0.950, 95% CI: 0.826–1.000) but substantially reduced sensitivity, F1 score, and MCC, indicating overly conservative classification behaviour.

Within the YOLOv11 family, models exhibited a greater tendency to prioritise sensitivity at the expense of specificity. According to the predefined composite score, YOLOv11-n achieved the highest overall ranking, providing the most balanced performance across the selected metrics. It combined high sensitivity (0.967, 95% CI: 0.917–1.000) with the highest specificity (0.350, 95% CI: 0.150–0.562), the highest F1 score (0.885, 95% CI: 0.816–0.940), and the highest MCC (0.434, 95% CI: 0.172–0.643) among all YOLOv11 variants. In contrast, the larger models (YOLOv11-l and YOLOv11-xl) achieved no statistically significant difference or even perfect sensitivity but showed markedly lower specificity and MCC, reflecting an increased tendency towards false-positive classifications. Notably, YOLOv11-xl classified all validation samples as myopic, resulting in zero specificity, an MCC of zero, and a non-estimable NPV, indicating a complete loss of discriminative ability for the non-myopic class despite its perfect sensitivity (see the confusion matrix in Supplementary Figure S3).

Overall, while several models achieved very high sensitivity, the joint consideration of sensitivity, MCC, and F1 score identified YOLOv8-m and YOLOv11-n as the most balanced and robust models on the internal validation dataset.

### 3.2. Statistical Comparison Between YOLOv8 and YOLOv11

Pairwise statistical comparisons between YOLOv8 and YOLOv11 models at the same architectural scale (n, s, m, l, and xl) were performed using paired cluster-based bootstrap resampling with Holm–Bonferroni correction for multiple testing. The results for the two primary outcome measures, sensitivity and MCC, are summarised in Table 2, which reports the mean performance differences (YOLOv8 − YOLOv11), corresponding 95% CIs, and adjusted *p*-values.

Pairwise comparisons between corresponding YOLOv8 and YOLOv11 variants revealed only two statistically significant differences after Holm–Bonferroni correction. First, YOLOv8-l showed substantially lower sensitivity than YOLOv11-l (Δ = −0.801, 95% CI: −0.897 to −0.698; adjusted *p* < 0.001), indicating that the YOLOv11 large variant was significantly more effective in identifying myopic eyes. Second, YOLOv8-xl achieved a significantly higher MCC than YOLOv11-xl (Δ = 0.416, 95% CI: 0.170–0.635; adjusted *p* < 0.05), reflecting a markedly better balance between true and false classifications. No significant differences were observed for the remaining comparisons in either sensitivity or MCC, suggesting that the overall performance of YOLOv8 and YOLOv11 was largely comparable across the nano, small, and medium variants. The significant advantage observed for YOLOv8-xl primarily reflects the preservation of discriminative ability for the non-myopic class, whereas YOLOv11-xl collapsed into predicting all validation samples as myopic, highlighting that increasing model complexity does not necessarily improve classification performance.

### 3.3. Independent Testing Phase

Table 3 shows the performance metrics of YOLOv8-m and YOLOv11-n on the independent test set, reported as mean values (central estimate) with corresponding 95% bootstrap CIs, including AUC values. Because the independent test set contained 70% myopic eyes, the reported precision (positive predictive value) and negative predictive value should be interpreted as being conditional on the prevalence of the study sample.

Confusion matrices for YOLOv8-m and YOLOv11-n, with TP, TN, FP, and FN values, are shown in the Supplementary Materials (Figures S4 and S5). YOLOv8-m and YOLOv11-n were further compared to experienced ophthalmologists.

**Figure 1:**
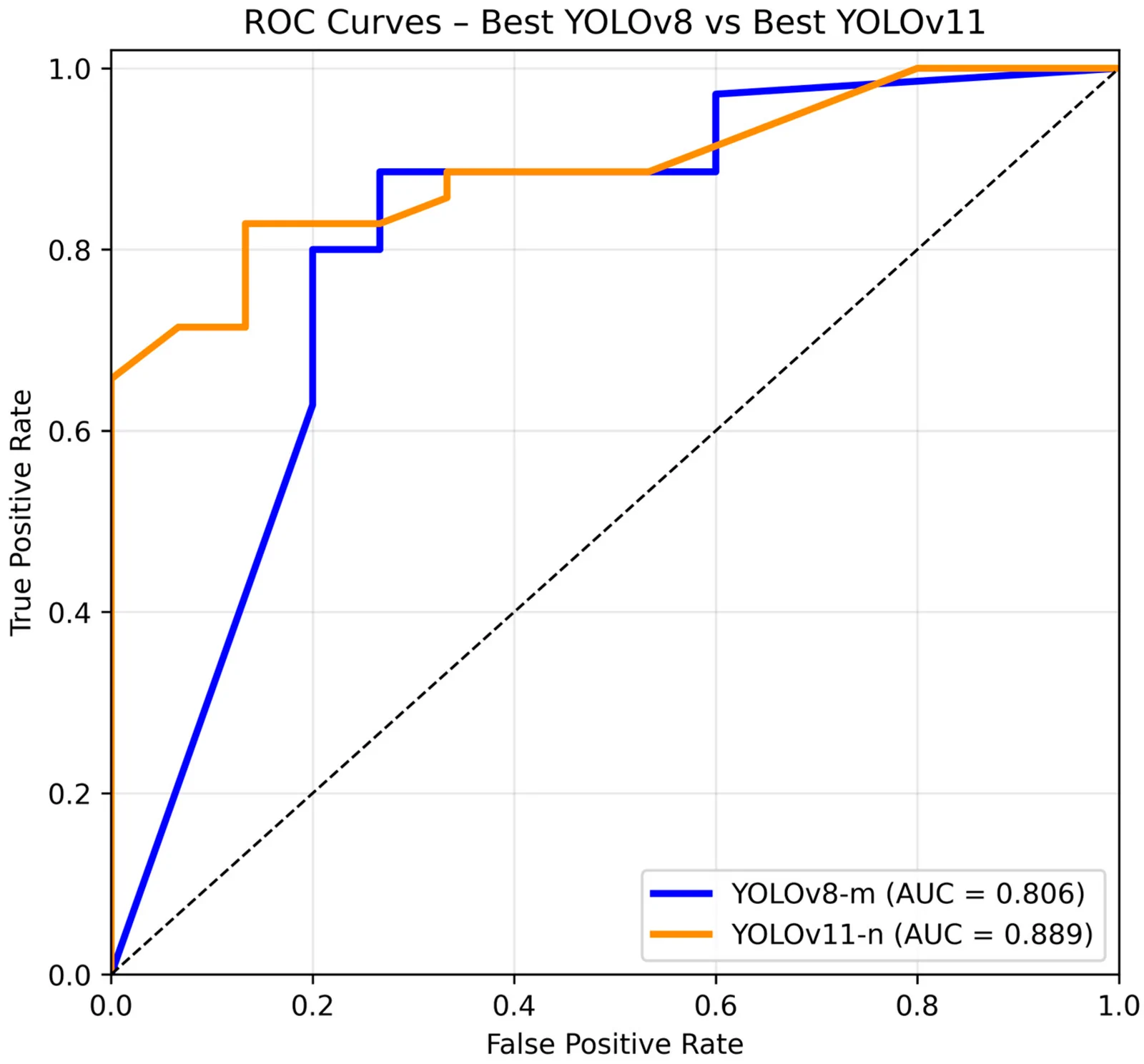
ROC curves and AUC values for YOLOv8-m and YOLOv11-n on independent test sets. The dashed line indicates a random classifier.

On the independent dataset, YOLOv8-m achieved an ROC AUC of 0.806, 95% CI: 0.653–0.941, whereas YOLOv11-n achieved an AUC of 0.889, 95% IC: 0.792–0.961. ROC curves for both models are shown in Figure 1.

To formally compare the discriminative ability of the two models, DeLong’s test for correlated ROC curves was applied. ROC curve analysis showed a numerically higher AUC for YOLOv11-n (0.889) than for YOLOv8-m (0.806). However, the DeLong test indicates that this difference was not statistically significant (ΔAUC = −0.083, *p* > 0.05), suggesting that the apparent improvement in discriminative performance may be attributable to sampling variability rather than to a true difference between the models.

To evaluate whether the two models differed significantly in their classification errors on the same images, a paired McNemar test was performed using binary predictions at the predefined operating threshold. McNemar’s exact test demonstrated no significant difference in classification between the two models (*p* > 0.05) on the independent test set.

### 3.4. Grad-CAM Activation Maps Interpretation

Grad-CAM analysis was performed to investigate spatial attention patterns used by YOLOv8-m and YOLOv11-n during prediction. In contrast to other studies [13,33], where specific regions of interest for myopia onset and myopia progression were identified, heat maps did not consistently converge on specific localised retinal regions. Instead, activation patterns were generally diffuse across the posterior pole, without clear and reproducible focal attention areas across images or across model architectures (Supplementary Figure S6). This behaviour was observed in both correctly and incorrectly classified cases and was consistent across YOLOv8-m and YOLOv11-n, limiting the interpretation of salient features contributing to correct and incorrect image classification.

### 3.5. Comparison with Expert Ophthalmologists

Five experienced ophthalmologists independently classified the same 50 fundus photographs from the independent test set based on visual inspection only. Performance metrics were computed for each observer and aggregated to estimate overall human diagnostic performance (Supplementary Table S2). Inter-observer agreement among the five ophthalmologists was fair, with a Fleiss’ κ of 0.383, indicating moderate variability in image interpretation. Pairwise agreement analysis using Cohen’s κ revealed fair to moderate agreement across most observer pairs, although one ophthalmologist consistently showed lower agreement with the remaining clinicians (Supplementary Table S3). Confusion matrices for all five ophthalmologists and the clinical consensus, including TP, TN, FP, and FN values, are presented in Supplementary Figure S7, while the corresponding expert ROC curves and AUC values are shown in Supplementary Figure S8.

**Figure 2:**
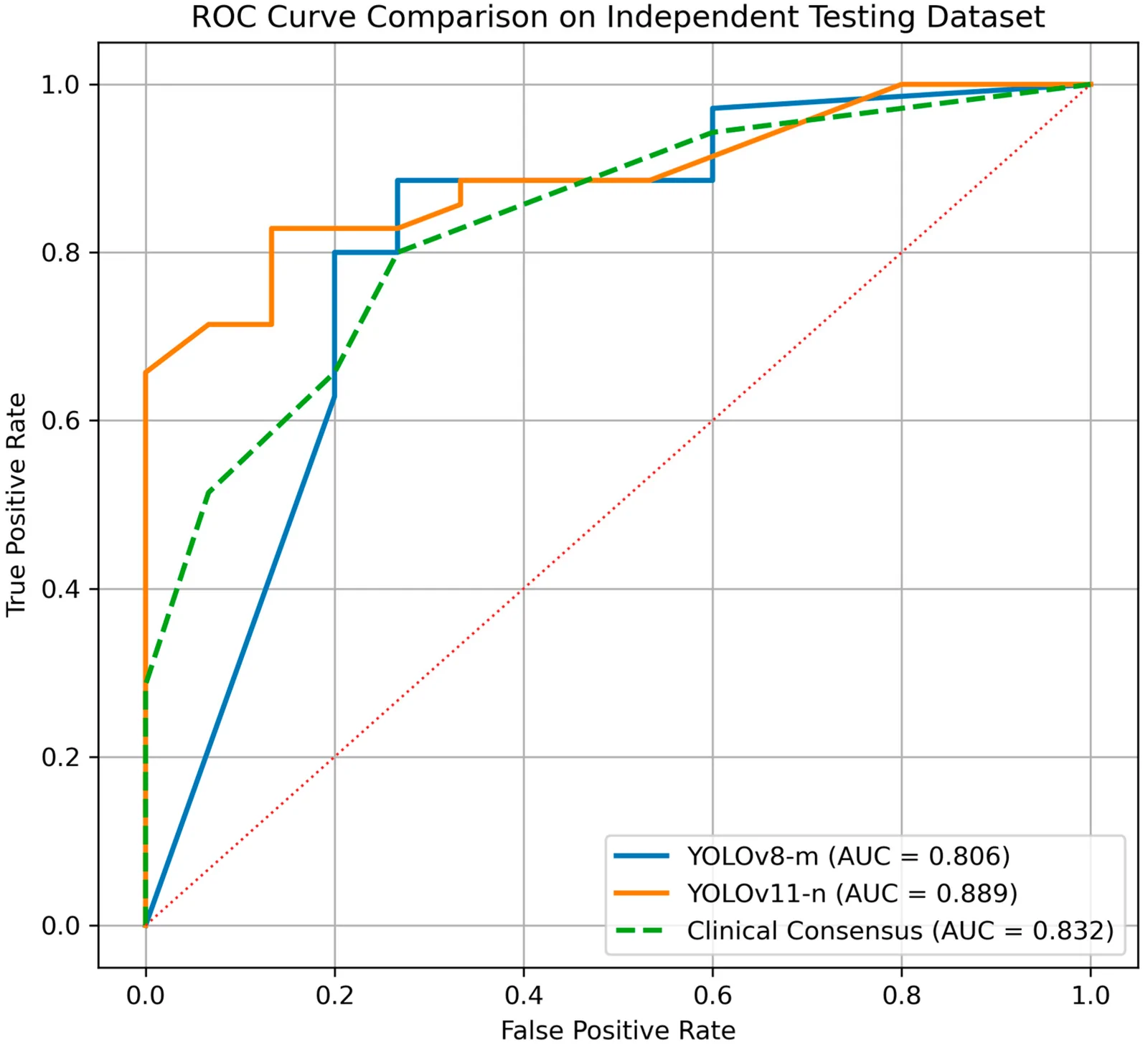
ROC curves for YOLOv8-m and YOLOv11-n compared to clinical consensus. AUC values are depicted in the bottom right corner. The red dotted line indicates a random classifier.

YOLOv8-m achieved an AUC of 0.806, while YOLOv11-n achieved an AUC of 0.889, compared with 0.832 for the clinical consensus derived from expert annotations (Figure 2).

ROC curve comparisons between the AI models and the clinical consensus were performed using DeLong’s test. The AUC difference between YOLOv8-m and the clinical consensus was −0.027 (*p* > 0.05), whereas the difference between YOLOv11-n and the clinical consensus was 0.056 (*p* > 0.05). No statistically significant differences in the AUC were observed between either AI model and the expert consensus.

Paired exact McNemar tests were performed to compare the classification decisions of each AI model with those of the clinical consensus. Table 4 reports the paired 2 × 2 contingency tables, including the concordant and discordant classification counts. In particular, ***b*** denotes cases correctly classified by the AI model but misclassified by the clinical consensus, and ***c*** denotes cases misclassified by the AI model but correctly classified by the clinical consensus. Neither YOLOv8-m (***b*** = 10, ***c*** = 5; exact McNemar *p* = 0.302) nor YOLOv11-n (***b*** = 9, ***c*** = 2; exact McNemar *p* = 0.065) showed statistically significant differences in paired classification outcomes compared with the clinical consensus. Although YOLOv11-n correctly classified a greater number of discordant cases than the clinical consensus, this trend did not reach statistical significance.

Pairwise comparisons between YOLOv8-m, YOLOv11-n, and the clinical consensus provided complementary insights into their diagnostic performance. Although YOLOv11-n achieved the highest numerical ROC AUC, DeLong’s test showed no statistically significant differences in discriminative ability compared with either YOLOv8-m or the clinical consensus. Likewise, the exact McNemar test revealed no statistically significant differences in paired classification outcomes between either AI model and the clinical consensus. Although YOLOv11-n correctly classified a greater number of discordant cases than the clinical consensus, this difference did not reach statistical significance. Overall, these findings indicate that the two selected YOLO models achieved classification performance that was not statistically different from that of the clinical consensus on the independent test set when evaluated at the predefined operating threshold. However, the study was not designed to establish equivalence or non-inferiority. A summary of diagnostic performance metrics is reported in Table 5.

## 4. Discussion

In this study, we evaluated the ability of YOLOv8 and YOLOv11 models to detect myopia from standard colour fundus photographs and compared their diagnostic performance with that of experienced ophthalmologists.

During internal validation, the YOLOv8 family demonstrated greater stability across architectural scales than the YOLOv11 family. Across both model families, sensitivity was consistently prioritised over specificity, reflecting a tendency to favour the detection of myopic eyes at the expense of an increased false-positive rate. This behaviour was particularly evident for the YOLOv11 variants and culminated in the YOLOv11-xl model, which classified all validation images as myopic, resulting in perfect sensitivity but zero specificity. Although the present study was not designed to investigate the underlying causes of this behaviour, it may reflect an excessive bias towards the majority class, potentially exacerbated by the limited sample size and class imbalance of the training dataset. In contrast, the YOLOv8 family showed a more consistent balance across model scales. However, only YOLOv8-l substantially shifted this trade-off towards higher specificity, achieving the highest specificity among all evaluated models at the expense of markedly reduced sensitivity. According to the predefined composite score, which was used exclusively for model selection, YOLOv8-m and YOLOv11-n emerged as the best-performing variants within their respective model families by providing the most balanced combination of sensitivity, MCC, and F1 score. Overall, YOLOv11 variants tended to achieve high sensitivity at the expense of lower specificity, whereas YOLOv8 models generally showed a more balanced trade-off between these metrics.

Evaluation on the independent test set showed that both YOLOv8-m and YOLOv11-n maintained good diagnostic performance when applied to previously unseen retinal fundus images from different patients. Although YOLOv11-n achieved the highest numerical ROC AUC (0.889), its advantage over YOLOv8-m (AUC = 0.806) was not statistically significant according to DeLong’s test. Likewise, no statistically significant differences in paired classification outcomes were observed between the two models, suggesting that the higher numerical AUC achieved by YOLOv11-n did not translate into a statistically demonstrable improvement in binary diagnostic performance at the predefined operating threshold. Taken together, these findings indicate that the selected models achieved no statistically significant difference in performance despite belonging to different model generations. Under the conditions of the present study, the architectural refinements introduced in YOLOv11 did not provide a measurable advantage over the YOLOv8-m model. More broadly, these results highlight the importance of distinguishing between numerical differences in performance metrics and statistically supported improvements in diagnostic performance. They also emphasise that good results obtained during internal validation should not be interpreted as evidence of superior clinical performance without confirmation on an independent test set. In relatively small single-centre clinical datasets, careful model selection, optimisation, and rigorous independent validation may therefore have a greater influence on diagnostic performance than increased architectural complexity alone.

The comparison between the AI models and the image-based assessment performed by experienced ophthalmologists should be interpreted within the specific experimental framework adopted in the present study. Participating ophthalmologists classified retinal fundus photographs without access to complementary clinical information, such as patient age, refractive error, visual acuity, ocular biometry, or medical history. Consequently, this comparison does not reflect comprehensive clinical diagnosis but rather evaluates the ability of both human observers and AI models to infer refractive status exclusively from retinal image features under identical information constraints. In routine clinical practice, the diagnosis and management of myopia rely on the integration of multiple clinical and biometric measurements. Therefore, the present findings should not be interpreted as evidence that image-based AI systems can replace comprehensive clinical assessment. Instead, they suggest that standard colour fundus photographs may contain discriminative image features associated with refractive status that may support automated image-based classification and justify further investigation of these models as decision support tools for myopia screening.

Comparison with the clinical consensus provided additional insights beyond conventional discrimination metrics. Although YOLOv11-n achieved the highest numerical ROC AUC (0.889), neither its comparison with YOLOv8-m nor with the clinical consensus revealed statistically significant differences in discriminative ability according to DeLong’s test. Likewise, the exact McNemar test showed no statistically significant differences in paired classification outcomes between either YOLOv8-m or YOLOv11-n and the clinical consensus on the small independent test set; however, the study was not designed to establish equivalence or non-inferiority. Although YOLOv11-n showed a numerical tendency to correctly classify more discordant cases than the clinical consensus, this difference did not reach statistical significance.

The fair level of inter-observer agreement observed among the five experienced ophthalmologists (Fleiss’ κ = 0.383) further highlights the inherent subjectivity of inferring refractive status from retinal fundus photographs alone. Pairwise Cohen’s κ analysis demonstrated generally fair to moderate agreement among most observer pairs, although one ophthalmologist consistently showed lower agreement with the remaining clinicians, contributing to the overall variability of the panel. These findings support the use of a majority-vote clinical consensus as the human reference standard and indicate that comparisons against a consensus panel may provide a more robust and clinically meaningful benchmark than comparisons with individual observers. Collectively, these findings suggest that the evaluation of AI systems for image-based myopia detection should consider not only overall discrimination but also agreement with expert clinical decision-making under realistic diagnostic conditions.

The clinical diagnosis of myopia is currently based on refractive error measurement, with cycloplegic refraction representing the gold standard. Retinal imaging is primarily used to detect myopia-related complications rather than to establish refractive diagnosis [10]. The present findings suggest that standard colour fundus photographs may contain discriminative image features associated with refractive status that can be identified by deep learning models. Although the underlying biological correlates remain to be fully elucidated, these results support the hypothesis that retinal structural and vascular characteristics may indirectly encode information related to ocular refractive status [13,19,34].

Rather than proposing fundus image analysis as a replacement for conventional refractive assessment, the present study supports its potential role as a complementary decision support approach. Such systems could be particularly valuable in large-scale screening programs, teleophthalmology services, and resource-limited settings where cycloplegic refraction, ocular biometry, or specialist assessment are not readily available. Importantly, the workflow adopted in this study relies exclusively on standard colour fundus photographs and a clinician-driven training strategy, demonstrating that reproducible AI models can be developed using widely available imaging devices without requiring advanced programming expertise. This may facilitate the adoption of similar workflows by eye care practitioners in both clinical and screening environments.

Most previous deep learning studies have focused on detecting pathological myopia or myopic maculopathy, conditions characterised by readily identifiable structural retinal abnormalities [17,18]. By contrast, the present study addressed the more challenging task of classifying refractive status in eyes without myopia-related pathology, where image differences are considerably subtler. This suggests that retinal fundus photographs may provide sufficient information to support automated identification not only of pathological myopia but also of non-pathological refractive status. Looking forward, refractive status classification could be integrated into comprehensive AI-based retinal analysis platforms capable of simultaneously detecting multiple ocular diseases and estimating refractive status from a single fundus photograph. Such multimodal decision support systems could enhance screening efficiency while providing clinicians with complementary information to prioritise referrals and guide subsequent diagnostic assessment.

Beyond the specific diagnostic task investigated, the present study demonstrates a reproducible clinician-oriented workflow for developing and validating computer vision models using widely available YOLO architectures. Rather than focusing solely on algorithmic innovation, this approach emphasises the translation of contemporary deep-learning frameworks into clinically relevant applications that can be implemented by eye care practitioners using standard retinal imaging and reproducible development pipelines. Such workflows may facilitate the broader adoption of AI-based image analysis in optometry and ophthalmology, particularly in settings where technical and computational resources are limited.

Grad-CAM analysis did not reveal consistent focal activation within specific retinal regions, with attention maps generally distributed across the posterior pole rather than concentrated in a single anatomical structure. Although Grad-CAM should not be interpreted as a direct representation of the features driving model predictions, this pattern suggests that refractive status classification may rely on distributed image characteristics rather than on localised retinal abnormalities [35,36]. Such image features may reflect underlying structural or vascular characteristics associated with refractive status, although this hypothesis cannot be confirmed by the present study. Alternatively, the diffuse activation patterns may reflect intrinsic limitations of post hoc explainability techniques when applied to complex, high-dimensional feature representations learned by deep neural networks. Consequently, the biological basis of AI-based refractive status prediction remains uncertain and warrants further investigation using complementary explainability approaches, multimodal retinal imaging, and feature attribution methods.

### Strengths and Limitations

The present study incorporates several methodological features designed to improve the robustness and clinical relevance of AI-based evaluation. First, we used a clinically defined refractive error as the reference standard, based on cycloplegic refraction measurements and the IMI definition [11,37]. To prevent data leakage, dataset partitioning was performed at the patient level before data augmentation, ensuring that both eyes from the same individual remained within the same subset. Data augmentation was applied exclusively to the training set. Model performance was evaluated using both an internal validation set and an independent held-out test set. Statistical robustness was further strengthened through patient-level paired cluster-based bootstrap analysis for the internal validation set, paired image-level bootstrap analysis for the independent test set, confidence interval estimation, and Holm–Bonferroni correction for multiple comparisons. The study also included direct benchmarking against experienced ophthalmologists performing blinded image-based assessments under identical information constraints. Furthermore, the adoption of a predefined clinically weighted composite model selection strategy, used exclusively during internal validation to identify the most balanced model within each architecture family, prioritising sensitivity, Matthews correlation coefficient, and F1 score, reflects diagnostic priorities relevant to myopia screening. Finally, the proposed workflow demonstrates the feasibility of developing clinically relevant deep learning models using widely available tools without requiring advanced programming expertise, thereby facilitating broader participation of eye care practitioners in AI-driven clinical research.

Several limitations should also be acknowledged. First, the study was based on a relatively small, single-centre dataset composed exclusively of Caucasian participants, with class imbalance, reflecting the patient population of a clinical practice primarily dedicated to myopia management. Therefore, performance metrics calculated after the testing phase, especially precision or PPV and NPV, should not be interpreted as estimates of expected predictive values in real-world screening settings. Although representative of the local clinical setting, these characteristics may have influenced model optimisation and limit the generalisability of the findings to broader populations. Second, although model performance was evaluated using an independent held-out test set comprising patients not included in model development, this cohort was also small and originated from the same clinical centre. Consequently, the reported performance estimates should be interpreted with appropriate caution because the limited sample size reduces statistical precision, and the retrospective single-centre design may have introduced unavoidable selection bias in both patient recruitment and test-set composition. Moreover, because all images were acquired using the same clinical environment and imaging protocol, the independent test set should not be considered a true external validation cohort. Consequently, the ability of the proposed models to generalise across different acquisition devices, clinical workflows, or geographically distinct populations remains to be established.

An additional limitation relates to the demographic characteristics of the study population. The non-myopic participants were older on average than the myopic group, making age a potential confounding factor. Since physiological retinal characteristics change throughout life, it cannot be excluded that part of the discriminative information learned by the models may reflect age-related retinal features in addition to structural characteristics associated with refractive status. Owing to the limited sample size, the present study was not designed to disentangle the independent contributions of age and refractive error to model performance. Consequently, it remains uncertain to what extent age-related retinal features contributed to the representations learned by the models. Future prospective multicentre studies including age-matched cohorts or appropriate statistical adjustment for age are warranted to clarify this issue.

Furthermore, only conventional colour fundus photographs were analysed, without integration of multimodal ocular imaging, biometric measurements, or additional clinical information. Likewise, although comparison with experienced ophthalmologists provided valuable clinical benchmarking, the number of participating experts was limited, and their assessments were intentionally restricted to fundus photographs without access to complementary clinical information. Model performance was evaluated using a predefined probability threshold, whereas alternative operating thresholds optimised for different clinical scenarios were not investigated. Therefore, this comparison reflects image-based classification performance rather than comprehensive clinical diagnosis. An additional methodological limitation is that model calibration was not formally evaluated. Calibration should be investigated in future studies.

Despite these limitations, the present study provides proof-of-concept evidence that the proposed clinician-driven workflows can extract potentially clinically relevant information associated with myopic refractive status from conventional colour fundus photographs. These findings support further investigation of image-based AI as a complementary decision support and screening tool, while emphasising that larger studies are required before consideration for routine clinical implementation.

Future research should therefore include large prospective multicentre studies with geographically diverse populations, age-matched cohorts or appropriate statistical adjustment for age, balanced class distributions, independent external validation across different imaging devices and clinical settings, or at least adding nested cross-validation if an external dataset is not available, integration of multimodal clinical data, formal evaluation of model calibration and explainability, investigation of the biological correlates underlying AI-based refractive status prediction, and assessment of clinician–AI collaboration within real-world screening workflows.

## 5. Conclusions

This proof-of-concept study demonstrated the feasibility of using YOLO-based computer vision models to classify myopic and non-myopic eyes from standard colour fundus photographs. Among the evaluated architectures, YOLOv8-m and YOLOv11-n demonstrated the most favourable overall balance between internal validation performance and independent testing, showing no statistically significant differences from the clinical consensus under controlled image-based evaluation conditions.

These findings support the feasibility of clinician-driven, no-code computer vision workflows for developing image-based decision-support tools that may complement large-scale screening and teleophthalmology programs, rather than replace comprehensive clinical assessment. However, these findings should be interpreted in the context of the relatively small single-centre dataset, class imbalance, demographic differences between study groups, and the absence of external multicentre validation. Larger prospective multicentre studies including geographically diverse, age-matched populations and independent external validation cohorts are required to confirm the robustness, generalisability, and clinical utility of both the proposed image classification models and the clinician-driven development workflow before consideration for routine clinical implementation.

Beyond the specific models evaluated, the present study demonstrates the feasibility of a clinician-driven workflow for developing and validating AI systems for retinal image analysis. Such a workflow may provide a reproducible framework for future research and facilitate broader participation of eye care practitioners in the development and clinical evaluation of computer vision applications.

## Figures and Tables

**Table 1:** Performance of YOLOv8 and YOLOv11 models on the internal validation dataset (mean and 95% bootstrap CIs).

Model	Accuracy	Sensitivity	Specificity	Precision	NPV	F1 Score	MCC	AUC
**YOLOv11-l**	0.763 (0.653–0.861)	0.967 (0.912–1.000)	0.150 (0.000–0.333)	0.773 (0.657–0.875)	0.600 (0.000–1.000)	0.859 (0.780–0.923)	0.209 (−0.079–0.458)	0.767 (0.620–0.892)
**YOLOv11-m**	0.750 (0.641–0.854)	0.917 (0.827–0.985)	0.250 (0.059–0.471)	0.786 (0.667–0.889)	0.500 (0.167–0.875)	0.846 (0.761–0.915)	0.218 (−0.039–0.480)	0.750 (0.610–0.876)
**YOLOv11-n**	0.813 (0.714–0.897)	0.967 (0.917–1.000)	0.350 (0.150–0.562)	0.817 (0.712–0.905)	0.778 (0.444–1.000)	0.885 (0.816–0.940)	0.434 (0.172–0.643)	0.748 (0.603–0.864)
**YOLOv11-s**	0.762 (0.653–0.859)	0.983 (0.944–1.000)	0.100 (0.000–0.250)	0.766 (0.654–0.865)	0.667 (0.000–1.000)	0.861 (0.781–0.923)	0.190 (−0.086–0.422)	0.781 (0.642–0.901)
**YOLOv11-xl**	0.750 (0.635–0.850)	1.000 (1.000–1.000)	0.000 (0.000–0.000)	0.750 (0.635–0.850)	not estimable	0.857 (0.777–0.919)	0.000 (0.000–0.000)	0.684 (0.549–0.820)
**YOLOv8-l**	0.362 (0.259–0.480)	0.167 (0.078–0.269)	0.950 (0.826–1.000)	0.909 (0.700–1.000)	0.275 (0.164–0.400)	0.282 (0.145–0.420)	0.147 (−0.039–0.282)	0.849 (0.717–0.951)
**YOLOv8-m**	0.812 (0.720–0.895)	0.967 (0.917–1.000)	0.350 (0.120–0.600)	0.817 (0.712–0.905)	0.778 (0.426–1.000)	0.885 (0.818–0.939)	0.434 (0.147–0.661)	0.757 (0.612–0.876)
**YOLOv8-n**	0.800 (0.711–0.883)	0.883 (0.800–0.955)	0.550 (0.308–0.773)	0.855 (0.750–0.939)	0.611 (0.350–0.833)	0.869 (0.796–0.926)	0.449 (0.186–0.668)	0.846 (0.734–0.929)
**YOLOv8-s**	0.775 (0.667–0.873)	0.967 (0.912–1.000)	0.200 (0.043–0.400)	0.784 (0.675–0.885)	0.667 (0.220–1.000)	0.866 (0.787–0.929)	0.274 (−0.008–0.520)	0.797 (0.680–0.901)
**YOLOv8-xl**	0.800 (0.707–0.887)	0.917 (0.833–0.981)	0.450 (0.250–0.682)	0.833 (0.733–0.922)	0.643 (0.375–0.882)	0.873 (0.800–0.932)	0.418 (0.172–0.640)	0.815 (0.696–0.910)

Values are reported as mean (95% CI). The mean value represents the estimate from the original dataset, before pairwise cluster-based bootstrapping. CIs were estimated using bootstrap resampling with 2000 iterations. NPV was not estimable for YOLOv11-xl because the classifier produced no negative predictions on the validation set. This can be verified from the confusion matrix in Supplementary Figure S3.

**Table 2:** Pairwise bootstrap comparison between YOLOv8 and YOLOv11 models on the internal validation dataset for sensitivity and MCC. Mean difference (YOLOv8-YOLOv11) and 95% CI, and adjusted *p*-value are reported.

Scale	Sensitivity Δ (95% CI)	Sensitivity p	MCC Δ (95% CI)	MCC p
**n**	−0.084 (−0.169–0.000)	0.160	0.012 (−0.200–0.232)	1.000
**s**	−0.016 (−0.076–0.036)	0.708	0.088 (−0.302–0.481)	1.000
**m**	0.050 (−0.018–0.125)	0.402	0.196 (−0.159–0.546)	0.988
**l**	−0.801 (−0.897– −0.698)	0.000	−0.062 (−0.356–0.237)	1.000
**xl**	−0.084 (−0.164– −0.017)	0.096	0.416 (0.170–0.635)	0.002

Mean differences (expressed by Δ) are reported as YOLOv8 minus YOLOv11 at the same model scale. CIs were obtained using paired bootstrap resampling (2000 iterations). *p*-values were adjusted for multiple comparisons using the Holm–Bonferroni method. Statistical significance was defined as adjusted *p* < 0.05.

**Table 3:** Performance of YOLOv8-m and YOLOv11-n on the independent test dataset (mean and 95% bootstrap CIs).

Model	Accuracy	Sensitivity	Specificity	Precision	NPV	F1	MCC	AUC
**YOLOv11-n**	0.840 (0.740–0.920)	0.829 (0.694–0.941)	0.867 (0.667–1.000)	0.935 (0.833–1.000)	0.684 (0.455–0.867)	0.879 (0.784–0.946)	0.656 (0.429–0.840)	0.889 (0.792–0.961)
**YOLOv8-m**	0.800 (0.680–0.900)	0.829 (0.687–0.946)	0.733 (0.500–0.938)	0.879 (0.750–0.972)	0.647 (0.400–0.882)	0.853 (0.746–0.935)	0.544 (0.274–0.783)	0.806 (0.653–0.941)

Values are reported as mean estimates (95% CI). CIs were estimated using bootstrap resampling with 2000 iterations.

**Table 4:** Paired McNemar test results comparing YOLO models with clinical consensus on the independent test set.

Comparison	*a*	*b*	*c*	*d*	Exact *p*-Value
**YOLOv8-m vs. Consensus**	30	10	5	5	0.302
**YOLOv11-n vs. Consensus**	33	9	2	6	0.065

***a*** indicates cases correctly classified by both AI and consensus; ***b*** indicates cases correctly classified by the AI model but misclassified by the clinical consensus; ***c*** indicates cases misclassified by the AI model but correctly classified by the clinical consensus; ***d*** indicates cases not correctly classified by both AI and consensus. Clinical consensus defined using majority voting (at least three concordant classifications out of five).

**Table 5:** Diagnostic performance of the two selected YOLO models and clinical consensus on the independent test set.

Model	Accuracy	Sensitivity	Specificity	Precision	NPV	F1	MCC	AUC
**YOLOv11-n**	0.840 (0.740–0.920)	0.829 (0.694–0.941)	0.867 (0.667–1.000)	0.935 (0.833–1.000)	0.684 (0.455–0.867)	0.879 (0.784–0.946)	0.656 (0.429–0.840)	0.889 (0.792–0.961)
**YOLOv8-m**	0.800 (0.680–0.900)	0.829 (0.687–0.946)	0.733 (0.500–0.938)	0.879 (0.750–0.972)	0.647 (0.400–0.882)	0.853 (0.746–0.935)	0.544 (0.274–0.783)	0.806 (0.653–0.941)
**Consensus**	0.700 (0.580–0.820)	0.657 (0.500–0.813)	0.800 (0.571–1.000)	0.885 (0.750–1.000)	0.500 (0.286–0.708)	0.754 (0.621–0.867)	0.420 (0.158–0.655)	0.832 (0.701–0.938)

Clinical consensus defined using majority voting (at least three concordant classifications out of five).

## Data Availability

The anonymised image dataset created for this research, along with the associated codes, trained models, image-level predictions, prediction scores, and statistical analysis code, are available from the corresponding author upon reasonable request, subject to institutional and ethical approval.

## Supplementary Materials

The following supporting information can be downloaded at https://www.mdpi.com/article/10.3390/jcm15166209/s1: Figure S1. Flow diagram describing participant selection, image quality assessment, and dataset partitioning; Figure S2: Confusion matrices for all YOLOv8’s variants after the internal validation phase: (a) nano, (b) small, (c) medium, (d) large, (e) extra large; Figure S3: Confusion matrices for all YOLOv11’s variants after the internal validation phase: (a) nano, (b) small, (c) medium, (d) large, (e) extra large; Figure S4: YOLOv8-m confusion matrix after the independent test phase; Figure S5: YOLOv11-n confusion matrix after the independent test phase; Figure S6: Grad-CAM visualizations for YOLOv8-m and YOLOv11-n are compared using pairs of retinal images and their corresponding heat maps. Examples a and c represent the same non-myopic eye correctly classified by YOLOv8-m (b) and YOLOv11-n (d), respectively. Examples e and g represent the same non-myopic eye not correctly classified by YOLOv8-m (f) but correctly classified by YOLOv11-n (h). In examples i and l, we show a non-myopic eye correctly classified by YOLOv11-n but with a completely different pattern, if compared to b and d, which limits the interpretability of common activation zones. The images in m and n, instead, depict an incorrectly classified non-myopic eye by YOLOv8-m with a completely different pattern than the one for the same condition in examples e and f. Even in this case, the difference in activation zones and pattern distribution limits the correct and precise interpretation of salient features contributing to image classification; Figure S7: Confusion matrices post-test for all expert ophthalmologists (from a to e) and the clinical consensus (f); Figure S8: ROC curves and AUC values for expert ophthalmologists on the independent test dataset. The diagonal dashed line represents a random classifier. Table S1: Summary description of patients’ characteristics and dataset details; Table S2: Expert ophthalmologists and calculated consensus performance metrics on the independent test dataset; Table S3: Inter-observer agreement among the five ophthalmologists. Overall agreement was fair (Fleiss’ κ = 0.383). Pairwise agreement is reported as Cohen’s κ.

## Abbreviations

The following abbreviations are used in this manuscript:

AI	Artificial Intelligence
AMP	Automatic Mixed Precision
DL	Deep Learning
YOLO	You Only Look Once
PNG	Portable Network Graphics
RGB	Red Green Blue colour space
HSV	Hue Saturation Value colour space
SER	Spherical Equivalent Refraction
CYL	Cylindrical power
D	Dioptre
IMI	International Myopia Institute
CSP	Cross Stage Partial
Grad-CAM	Gradient Weighted Class Activation Mapping
ACC	Accuracy
P	Precision
S	Sensitivity
Sp	Specificity
ROC	Receiver Operating Characteristic curve
AUC	Area Under the Curve
TPR	True Positive Rate
FPR	False Positive Rate
TP	True Positive
TN	True Negative
FP	False Positive
FN	False Negative
MCC	Matthew Correlation Coefficient
NPV	Negative Predictive Value
CIs	Confidence Intervals
