# Supplementary material for: Detection of Myopia from Colour Fundus Photographs Using YOLO-Based Computer-Vision Models: A Comparison with Expert Ophthalmologists
Source: J Clin Med. 2026 Aug 11;15(16):6209. doi: 10.3390/jcm15166209 (PMC13513927; doi:10.3390/jcm15166209)
Supplement: Supplementary file 1 [file jcm-15-06209-s001.zip › jcm-4464558-supplementary.pdf]

Table S1. Summary description of patients' characteristics and dataset details.

**Characteristics**	**Myopic**	**Non-Myopic**	**Total**
Participants	124 (74 F, 50 M)	45 (27 F, 18 M)	169
Sex (%)	59.7 F/40.3 M	60.0 F/40.0 M	59.8 F/40.2 M
Prevalence in sample (%)	73.4	26.6	---
**Participants after image quality assessment**	119 (71 F, 48 M)	43 (25 F, 18 M)	162
Images after quality assessment (n)	238	86	324
Mean age (SD)	26.74 (13.78)	40.33 (17.74)	30.38 (16.07)
Median age (IQR)	25 (15-34)	44 (28-53)	28.5 (16-43)
Age range (min-max)	from 9 to 61	from 7 to 84	from 7 to 84
Mean SER (SD) D	-3.19 (2.84)	+0.92 (1.32)	-2.10 (3.11)
SER range (min-max) D	-8.00 to -0.50	-0.38 to +6.50	-8.00 to +6.50
N. Eyes with high myopia (≤-6.00 D)	20 (8.40%)	-	-
Data collection period	March-August 2023	Idem	Idem
**Dataset Composition**	**Training set**	**Validation set**	**Independent set**
Patients (n)	122	40	50
Eyes (n)	244	80	50
Myopic patients (n)	89	30	35
Non-myopic patients (n)	33	10	15
Myopic eyes (n)	178	60	35
Non-myopic eyes (n)	66	20	15

F: female; M: male; SD: standard deviation; IQR: interquartile range; D: dioptre; SER: spherical equivalent refraction.

Table S2. Expert ophthalmologists and calculated Consensus performance metrics on the independent test dataset.

**Observer**	**Accuracy**	**Sensitivity**	**Specificity**	**Precision**	**F1**	**NPV**	**MCC**	**AUC**
Doctor_1	0,660	0,629	0,733	0,846	0,721	0,458	0,332	0,688
Doctor_2	0,800	0,743	0,933	0,963	0,839	0,609	0,622	0,870
Doctor_3	0,660	0,686	0,600	0,800	0,738	0,450	0,267	0,613
Doctor_4	0,560	0,353	1,000	1,000	0,522	0,421	0,385	0,673
Doctor_5	0,740	0,800	0,600	0,824	0,812	0,562	0,393	0,706
Consensus	0,700	0,657	0,800	0,885	0,754	0,500	0,420	0,832

Clinical consensus, defined using majority voting (at least three concordant classifications out of five). NPV: negative predictive value; F1: F1 score; MCC: Matthews correlation coefficient; AUC: area under the receiver operating characteristic curve.

Table S3. Inter-observer agreement among the five ophthalmologists. Overall agreement was fair (Fleiss' κ = 0.383). Pairwise agreement is reported as Cohen's κ.

	**Doctor 1**	**Doctor 2**	**Doctor 3**	**Doctor 4**	**Doctor 5**
**Doctor 1**	1.000	0.478	0.516	0.295	0.594
**Doctor 2**	0.478	1.000	0.390	0.347	0.547
**Doctor 3**	0.516	0.390	1.000	0.275	0.397
**Doctor 4**	0.295	0.347	0.275	1.000	0.191
**Doctor 5**	0.594	0.547	0.397	0.191	1.000

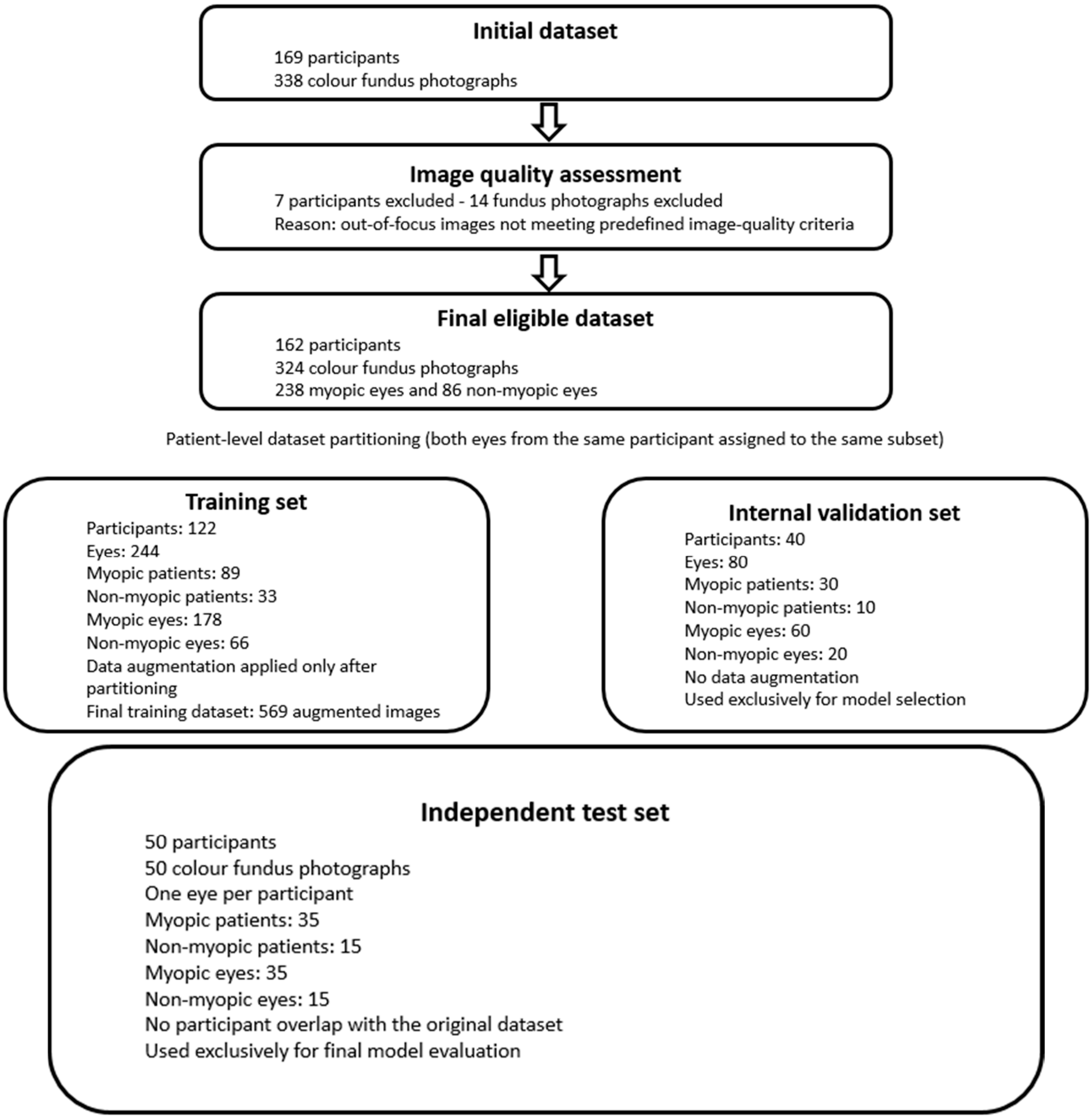

Figure S1. Flow diagram describing participant selection, image quality assessment, and dataset partitioning.

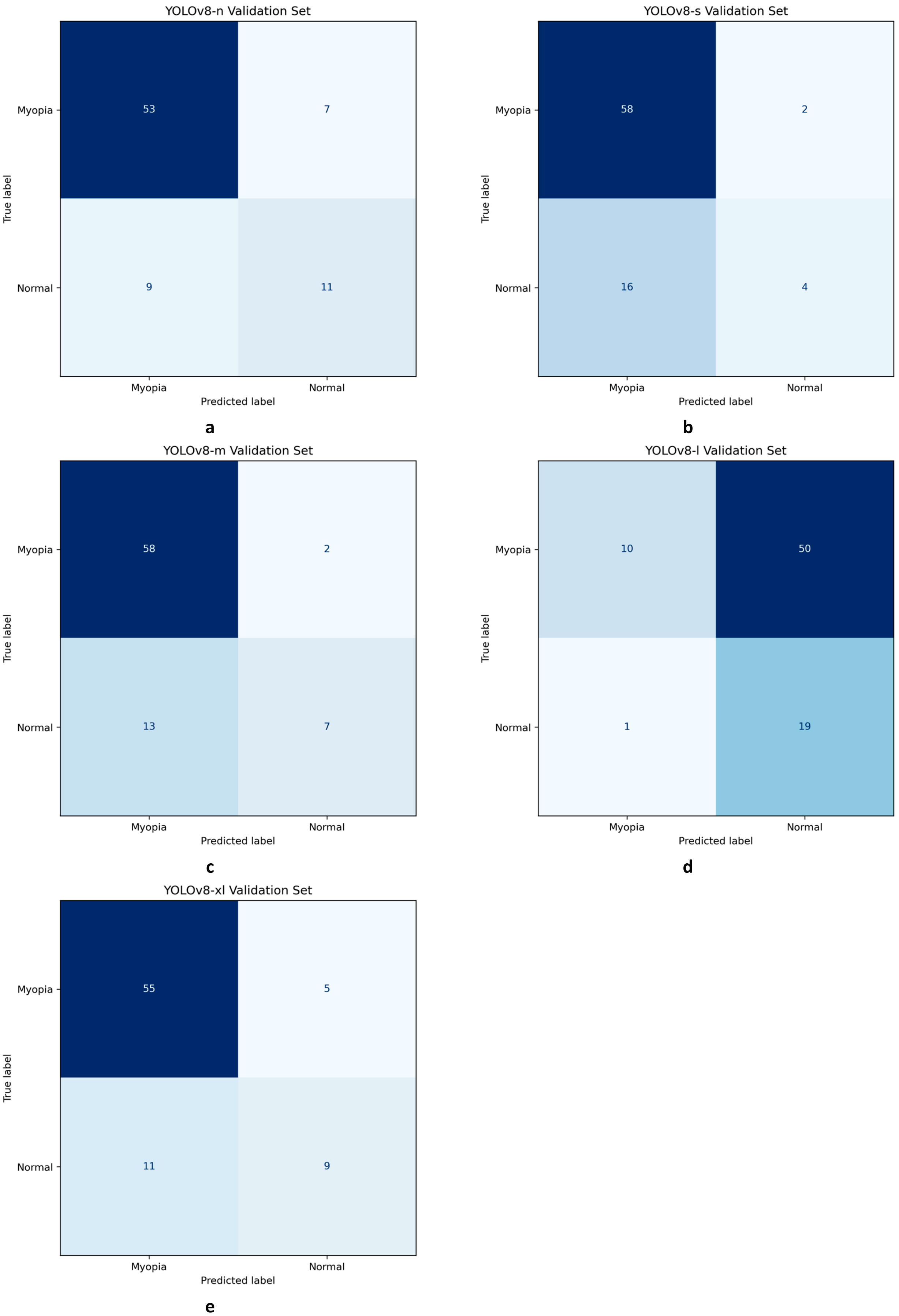

Figure S2 Confusion matrices for all YOLOv8 variants after the internal validation phase: **a**. nano, **b**. small, **c**. medium, **d**. large, **e**. extra-large.

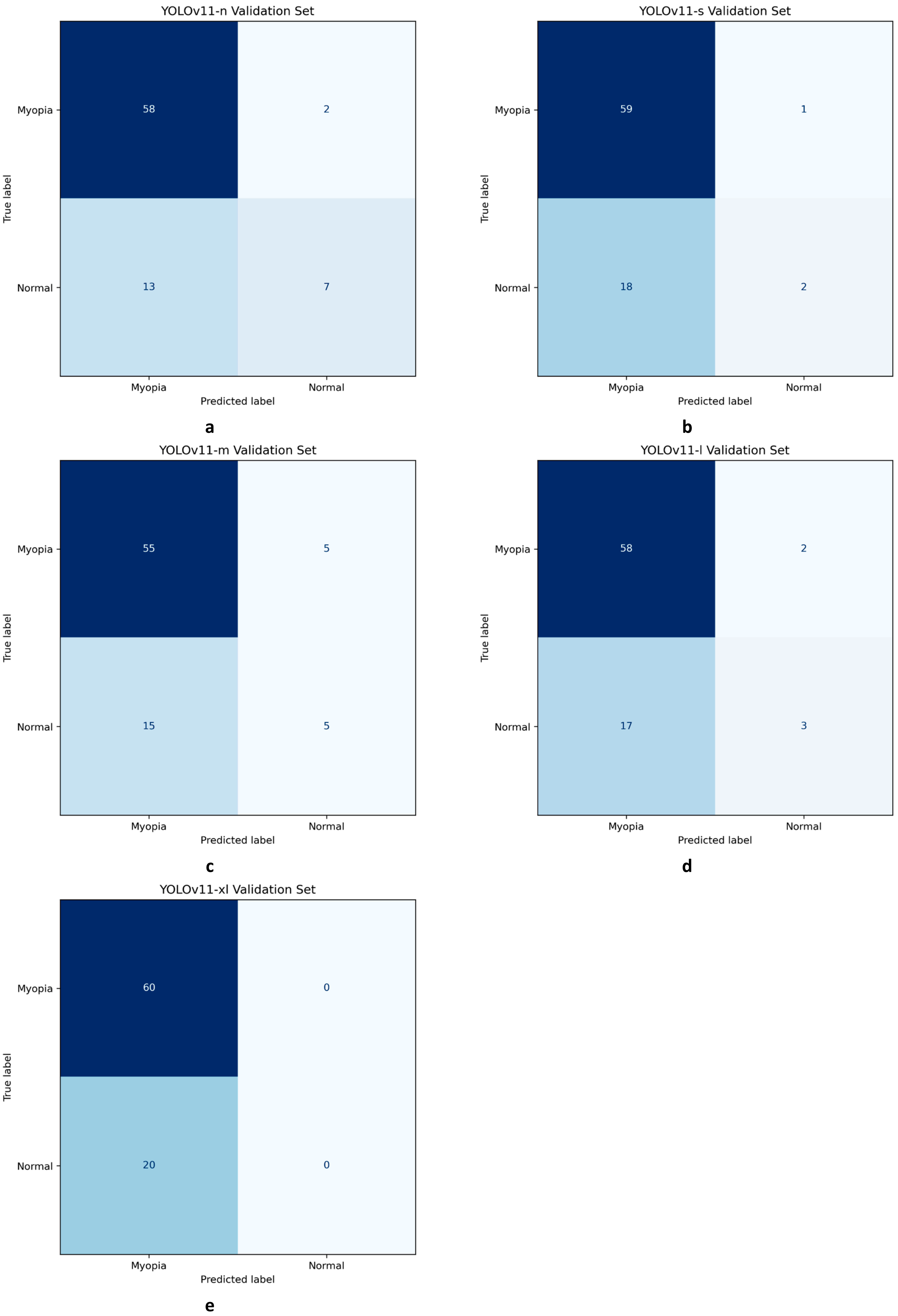

Figure S3 Confusion matrices for all YOLOv11 variants after the internal validation phase: **a**. nano, **b**. small, **c**. medium, **d**. large, **e**. extra-large.

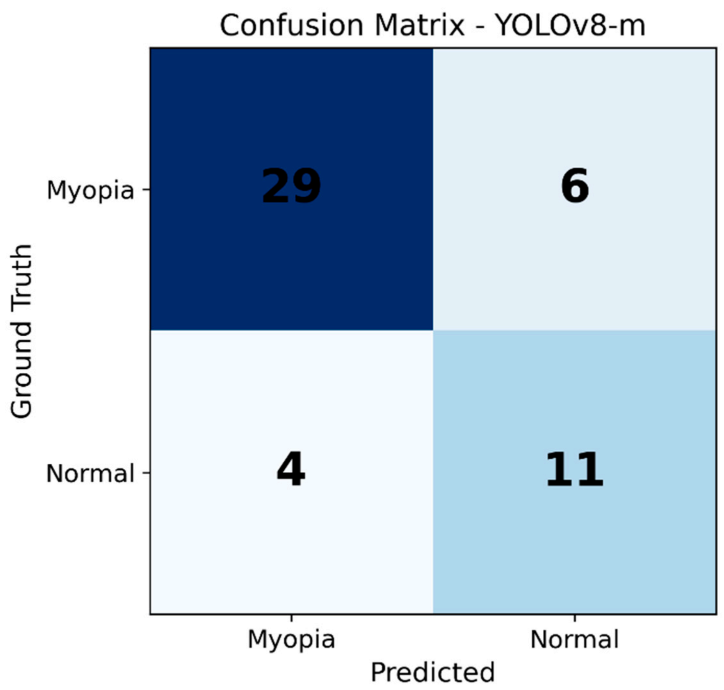

Figure S4 YOLOv8-m confusion matrix after the independent test phase.

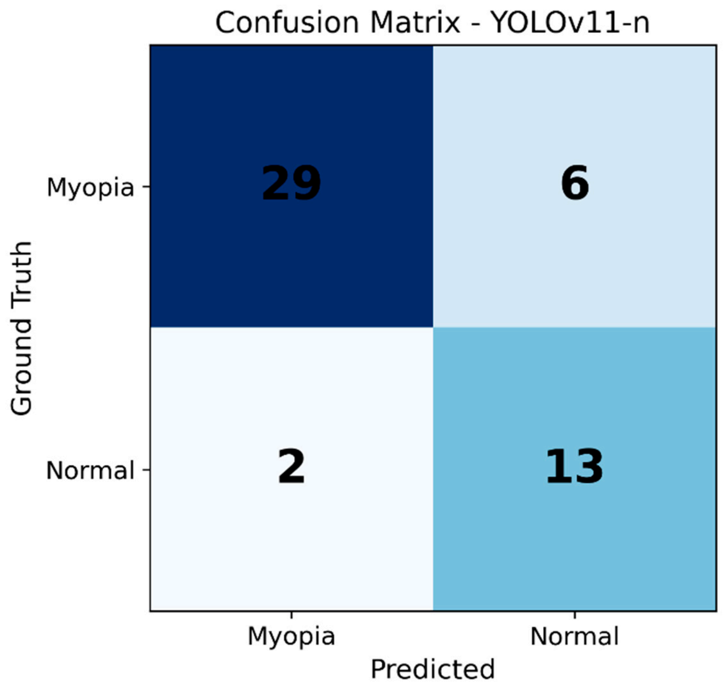

Figure S5 YOLOv11-n confusion matrix after the independent test phase.

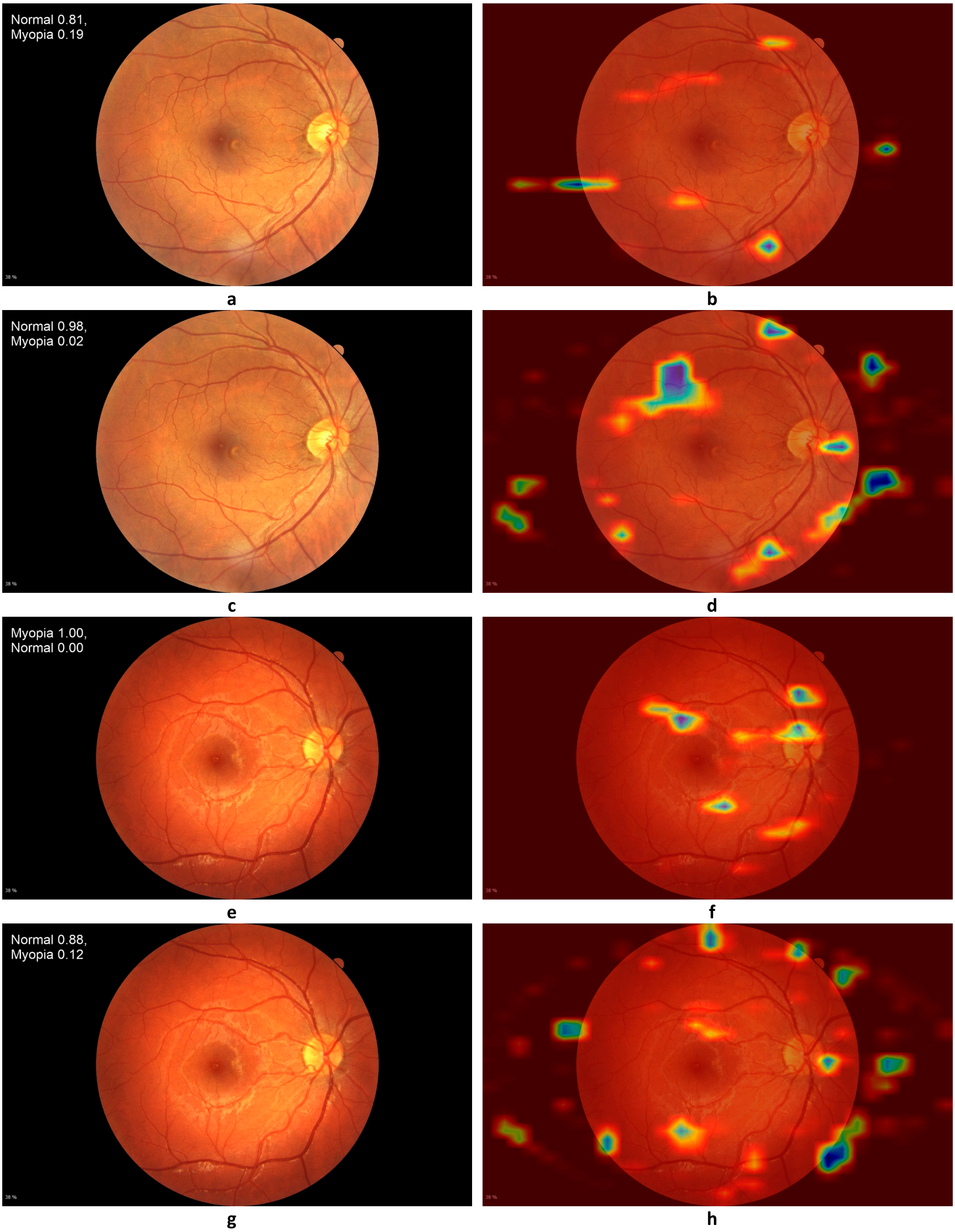
Normal 0.81,
Myopia 0.19
a
b
Normal 0.98,
Myopia 0.02
c
d
Myopia 1.00,
Normal 0.00
e
f
Normal 0.88,
Myopia 0.12
g
h

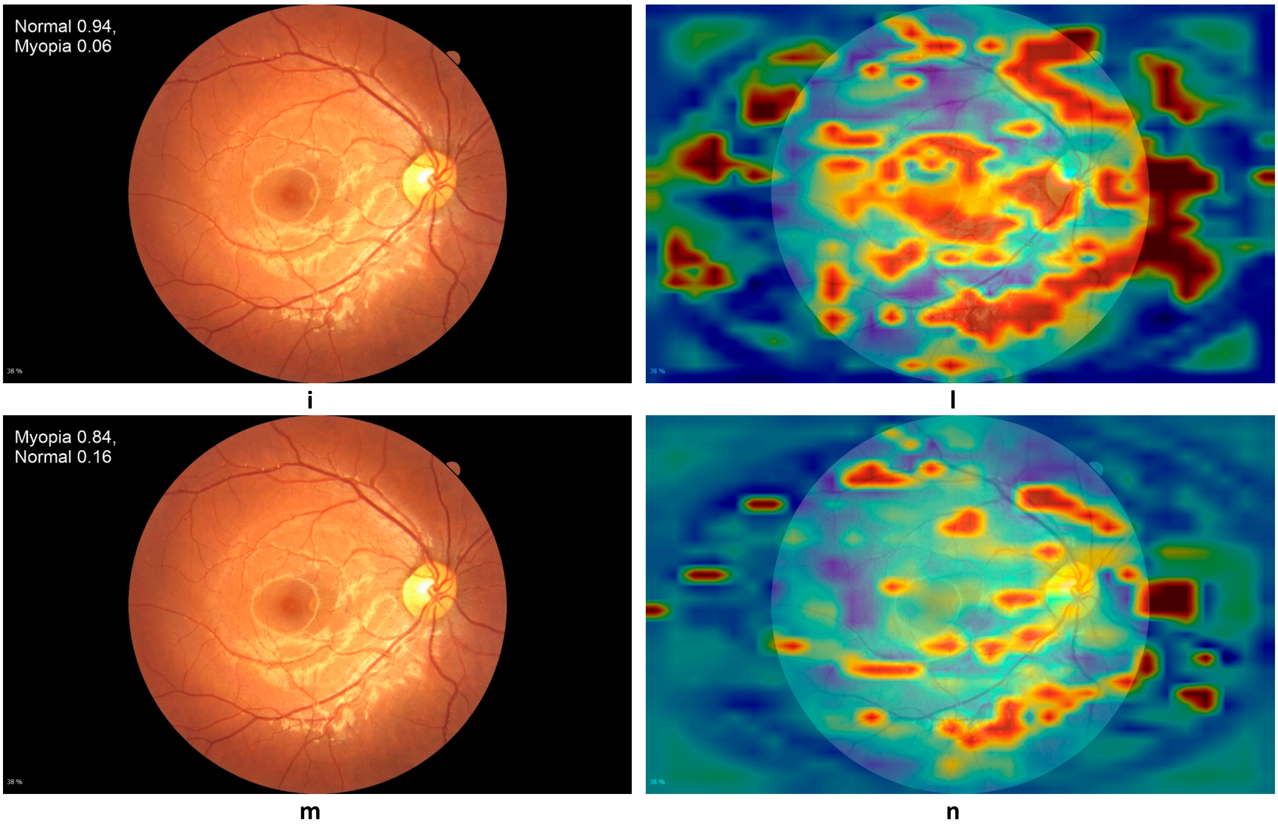

Figure S6. Grad-CAM visualizations for YOLOv8-m and YOLOv11-n are compared using pairs of retinal images and their corresponding heat-maps. Examples **a** and **c** represent the same non-myopic eye correctly classified by YOLOv8-m (**b**) and YOLOv11-n (**d**), respectively. Examples **e** and **g** represent the same non-myopic eye not correctly classified by YOLOv8-m (**f**) but correctly classified by YOLOv11-n (**h**). In examples **i** and **l,** we show a non-myopic eye correctly classified by YOLOv11-n but with a completely different pattern, if compared to **b** and **d**, which limits the interpretability of common activation zones. The images in **m** and **n**, instead, depict an incorrectly classified non-myopic eye by YOLOv8-m, with a completely different pattern than the one for the same condition in examples **e** and **f**. Even in this case, the difference in activation zones and pattern distribution limits the correct and precise interpretation of salient features contributing to image classification.

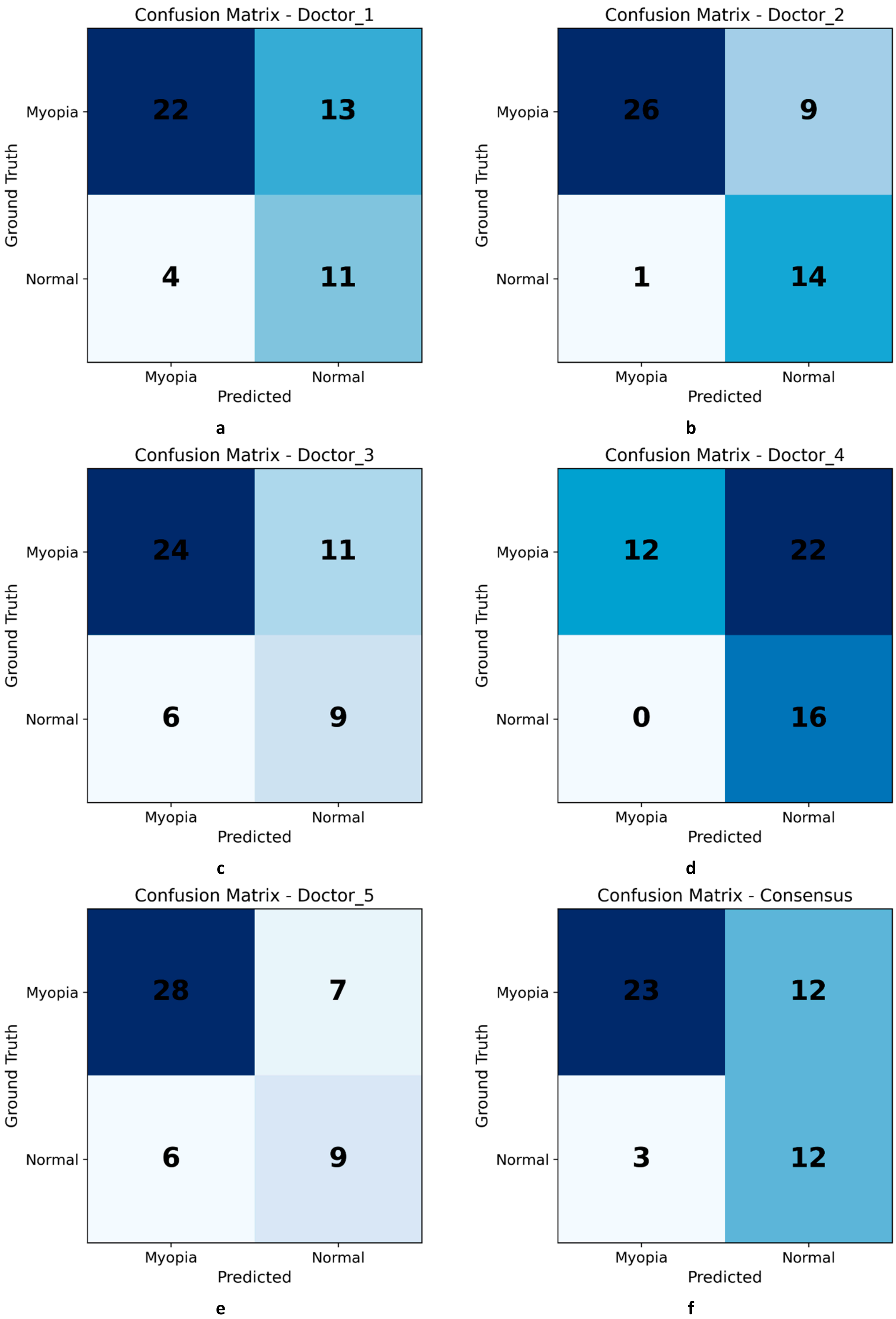

Figure S7. Confusion matrices post-test for all expert ophthalmologists (from **a** - **e**) and the clinical consensus (**f**).

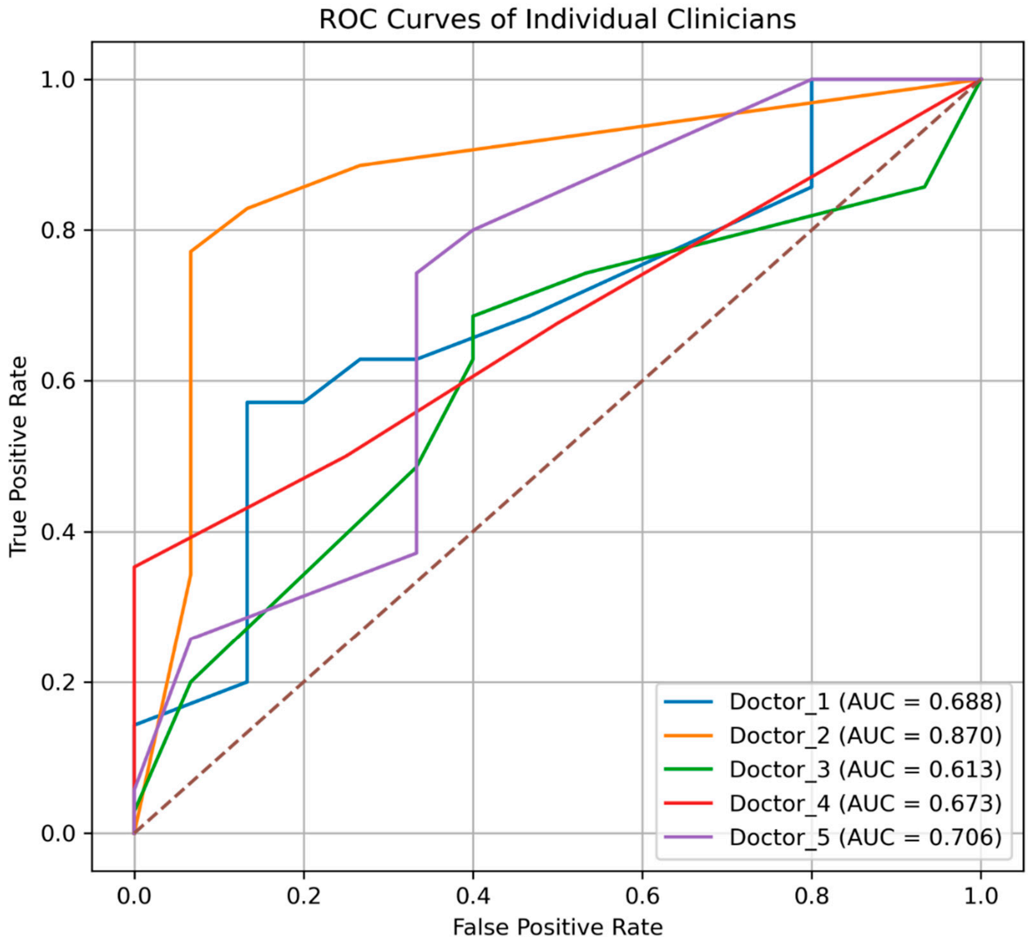

Figure S8. ROC curves and AUC values for expert ophthalmologists on the independent test dataset. The diagonal dashed line represents a random classifier.
